# Berberine Suppresses TNF-α-induced MMP-9 and Cell Invasion through Inhibition of AP-1 Activity in MDA-MB-231 Human Breast Cancer Cells

**DOI:** 10.3390/molecules13122975

**Published:** 2008-12-03

**Authors:** Sangmin Kim, Jae Hyuck Choi, Jong Bin Kim, Seok Jin Nam, Jung-Hyun Yang, Jung-Han Kim, Jeong Eon Lee

**Affiliations:** 1 Department of Surgery, Samsung Medical Center, Sungkyunkwan University School of Medicine, 50 Ilwon-dong, Kangnam-gu, Seoul, South Korea, 135-710; 2 Cancer Research Institute, Seoul National University College of Medicine, 28 Yongon-dong, Chongno-gu, Seoul, South Korea

**Keywords:** Berberine, AP-1, MMP-9, Cell invasion

## Abstract

Invasion of cancer cell induced by matrix metalloproteinase-9 (MMP-9) is one of pivotal steps in cancer metastasis. Herein, we investigated how cell invasion was regulated by berberine (BBR), an isoquinoline derivative alkaloid compound, in MDA-MB-231 human breast cancer cells. The basal level of MMP-9 activity and expression was dose-dependently increased by TNF-α, while TNF-α-induced MMP-9 gelatinase activity and expression was decreased by BBR. To investigate regulatory mechanism of TNF-α-induced MMP-9 expression, we pretreated cells with UO126 (MEK inhibitor), SB203580 (p38 inhibitor) and SP600125 (JNK inhibitor), respectively. Interestingly, TNF-α-induced MMP-9 activity and expression was decreased by UO126 and SB203580, but not by SP600125. Therefore, we further examined the effects of BBR on TNF-α-induced AP-1 DNA binding activity which is a downstream target of ERK and p38. Our data showed that TNF-α-induced AP-1 DNA binding activity was inhibited by BBR. Finally, we investigated the effect of BBR on TNF-α-induced cell invasion. TNF-α-induced cell invasion was significantly decreased by BBR treatment. Taken together, we suggest that TNF-α-induced MMP-9 expression and cell invasion are decreased by BBR through the suppression of AP-1 DNA binding activity in MDA-MB-231 human breast cancer cells.

## Introduction

One of the general properties of cancer is the ability to invade surrounding tissues and metastasize to other organs [1]. Tumor invasion requires degradation of the extracellular matrix (ECM) proteins and cell migration [2]. Lysis of ECM by specific proteinases including MMP-2 and MMP-9 need to cell invasion [1]. Therefore, MMPs can be a promising therapeutic target in cancer treatment [3].

Matrix metalloproteinases (MMPs) are a major group of enzymes which regulate cell-matrix composition [4]. MMPs are a family of 24 human zinc-binding endopeptidases which can degrade virtually all extracellular matrix (ECM) components [5]. These MMPs are synthesized in most cells and immediately secreted into the ECM [6]. They play an important role in various cellular processes including embryogenesis, inflammation, wound healing, arthritis, cardiovascular disease and cancer invasion [4]. In those MMPs, MMP-9 (92 kDa gelatinase) is particularly known to play a critical role in cancer progress, such as angiogenesis as well as tumor growth, invasion and distant metastasis of various tumors and breast cancer as well [7,8].

MMP-9 belongs to the gelatinase subgroup of the MMP family and its expression and activity have been associated with different stages of carcinoma progression [9]. MMP-9 promoter activity is mediated by several transcription factor-binding motifs including AP-1 and nuclear factor-κB (NF-κB) consensus sites [10]. Activation of JNK1 and c-Jun stimulates MMP-9 expression via AP-1 at -79 bp [11].

Berberine is an isoquinoline derivative alkaloid that has been isolated from *Berberis aquifolium* (Oregon grape), *Berberis aristata* (tree turmeric), *Berberis vulgaris* (barberry) and *Hydrastis canadensis* (goldenseal) [12]. Berberine, the major ingredient in these herbs, has many pharmacological effects, including inhibition of DNA and protein synthesis, cell cycle arrest and anti-cancer effects [13,14]. In our previous study, we reported that berberine suppresses TPA-induced MMP-9 expression in human primary keratinocytes [15]. In addition, basal and UV-induced MMP-1 expression is known to be inhibited by berberine in human dermal fibroblasts [16].

The aim of this investigation was to evaluate the effect of berberine on MMP-9 expression and tumor cell invasion. We found that berberine suppresses TNF-α-induced MMP-9 expression and cell invasion and also inhibited TNF-α-induced AP-1 DNA binding activity which was regulated by Jun and Fos families. With further pathway analysis, we demonstrated berberine antagonizes TNF-α-induced MMP-9 expression and cell invasion by TNF-α through inactivation of AP-1 pathway in MDA-MB-231 cells.

## Results and Discussion

### Dose-dependent inhibition of TNF-α-induced MMP-9 gelatinase activity/expression by berberine

**Figure 1:**
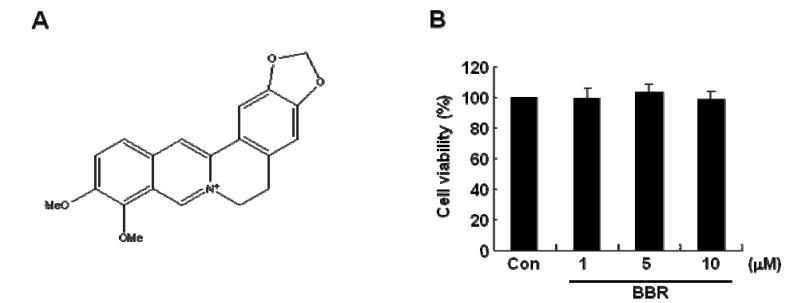
The viability of MDA-MB-231 cells with treatment of berberine in different concentration. After serum-starvation for 16 h, cells treated with berberine at the indicated concentration for 48 h in fresh serum-free media. (A) Berberine structure. (B) Cell viabilities were analyzed by MTT assay as described in Materials and Methods. These results were representative of three independent experiments. Values shown are means ± SEM. Con; control.

To verify the effect of BBR on cell viabilities, we treated cells with BBR at the indicated concentration for 24 h. The structure of berberine is given in Figure 1A. Cell viabilities were not affected by BBR in the indicated concentration (Figure 1B).

**Figure 2:**
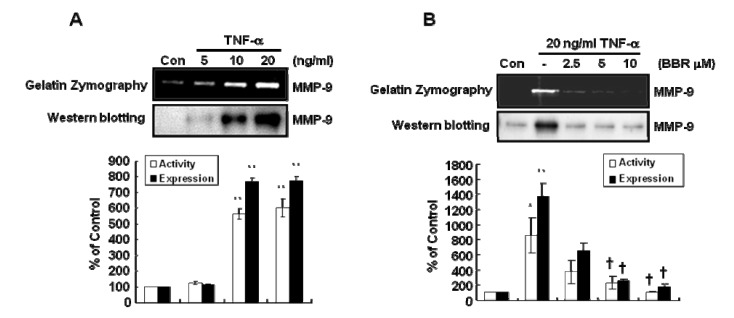
TNF-α-induced MMP-9 activity and expression are dose-dependently decreased by berberine in MDA-MB-231 cells. (A) After serum-starvation for 16 h, cells were treated with TNF-α (20 ng/ml) for 24 h. (B) After serum-starvation for 16 h, cells were pretreated with berberine at the indicated concentration for 1 h and then treated with TNF-α (20 ng/ml) for 24 h. MMP-9 activity and expression were analyzed by Zymography (upper band) and Western blotting (lower band), respectively. These results were representative of three independent experiments. Values shown are means ± SEM. * *P* < 0.05, **P < 0.01 *vs*. control, † *P* < 0.05 *vs*. TNF-α-treated cells. Con; control.

To examine the effect of BBR on TNF-α-induced MMP-9 gelatinase activity and expression, we treated cells with TNF-α at the indicated concentration for 24 h in serum-free media. The level of MMP-9 gelatinase activity (upper band) and expression (lower band) were dose-dependently increased by TNF-α (Figure 2A). The level of MMP-9 gelatinase activity (upper band) and expression (lower band) by TNF-α treatment was significantly increased by 601 ± 58.3% and 773.6 ± 28.5% of control level, respectively, at the concentration of 20 ng/mL TNF-α. On the other hand, TNF-α-induced MMP-9 gelatinase activity (upper band) and expression (lower band) were decreased by BBR treatment in a dose-dependent manner (Figure 2B). TNF-α-induced MMP-9 gelatinase activity (upper band) and expression (lower band) were significantly increased by 1005.4±234.8%and 1707.2±168.8% of control level, respectively, whereas TNF-α-induced MMP-9 gelatinase activity and expression were decreased by 104.2±3.0% and 244.2± 31.9% of control level at the concentration of 10 μM BBR treatment. Based on these results, we suggest that TNF-α-induced MMP-9 gelatinase activity/expression is suppressed by BBR treatment in MDA-MB-231 cells.

### Decrease of TNF-α-induced MMP-9 gelatinase activity/expression by Bay11-7086, UO126 and SB203580

**Figure 3:**
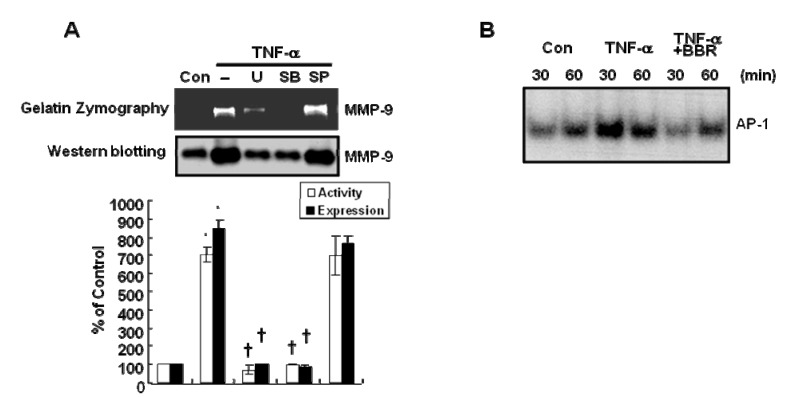
TNF-α-induced MMP-9 activity and expression was inhibited by MEK and p38 inhibitors and TNF-α-induced AP-1 DNA binding activity were suppressed by berberine in MDA-MB-231 cells. (A) After serum-starvation for 16 h, cells were pretreated with 10 μM UO126, SB203580 and SP600126, respectively, for 30 min and then treated with 20 ng/mL TNF-α for 24 h. MMP-9 activity and expression were analyzed by Zymography (upper band) and Western blotting (lower band), respectively. (B) After serum-starvation for 16 h, cells were pretreated with 10 μM berberine for 1 h and then treated with 20 ng/mL TNF-α for the indicated times. (B) Using the nucleus protein, AP-1 DNA binding activity was analyzed by EMSA as described in Materials and Methods. These results were representative of three independent experiments. Values shown are means ± SEM. * *P* < 0.01 *vs*. control. † *P* < 0.01 *vs*. TNF-α-treated cells.Con; control, U; UO126, SB; SB203580, SP; SP600126.

To investigate the mechanism of regulation of MMP-9 gelatinase activity/expression by TNF-α, we pretreated cells with various inhibitors such as MEK inhibitor, UO126, JNK inhibitor, SP600125 and p38 inhibitor, SB203580 at the indicated concentration prior to 20 ng/mL TNF-α treatments. After 24 h, culture media were harvested and subjected to Zymography and MMP-9 gelatinase activity and expression then detected. Our results showed that TNF-α-induced MMP-9 gelatinase activity (upper band) and expression (lower band) are suppressed by UO126 and SB203580, respectively (Figure 3A). The level of MMP-9 gelatinase activity and expression increased significantly by 738.5±40.5% and 845.0± 46.0%, respectively, at the concentration of 20 ng/mL TNF-α treatment. On the other hand, TNF-α-induced MMP-9 gelatinase activity was decreased significantly by 96.2±3.9% and 107.0±3.0%, respectively, at the concentration of 10 μM UO126 and SB203580 treatment(Figure 3A upper band). In addition, TNF-α-induced MMP-9 expression was decreased significantly by 99.5±2.5% and 118.1±7.9%, respectively, at the concentration of 10 μM UO126 and SB203580 treatment (Figure 3A lower band). However, TNF-α-induced MMP-9 gelatinase activity/expression were not affected by JNK inhibitor, SP600125. Therefore, we suggest that TNF-α-induced MMP-9 expression and activity are regulated by ERK and p38-dependent pathway in MDA-MB-231 cells.

### Inhibition of TNF-α-induced AP-1 DNA binding activity by berberine

Next, we investigated the effect of BBR on TNF-α-induced AP-1 DNA binding activity in MDA-MB-231 cells. In previous studies, MMP-9 promoter contains cis-acting elements for transcription factors that include two AP-1 sites and an NF-κB site and also MMP-9 expression is up-regulated by transcriptional activities of AP-1 and NF-κB [10,18]. In accordance with these reports, we investigated the effect of BBR on TNF-α-induced AP-1 DNA binding activity in MDA-MB-231 cells. We pretreated cells with 10 μM BBR for 1 h prior to 20 ng/mL TNF-α treatment at the indicated times. We observed that TNF-α-induced AP-1 DNA binding activity is decreased by BBR (Figure 3B). Therefore, we suggest that BBR suppresses TNF-α-induced MMP-9 expression by inhibition of AP-1 DNA binding activity in MDA-MB-231 cells.

### Prevention of TNF-α-induced cell invasion by berberine

**Figure 4:**
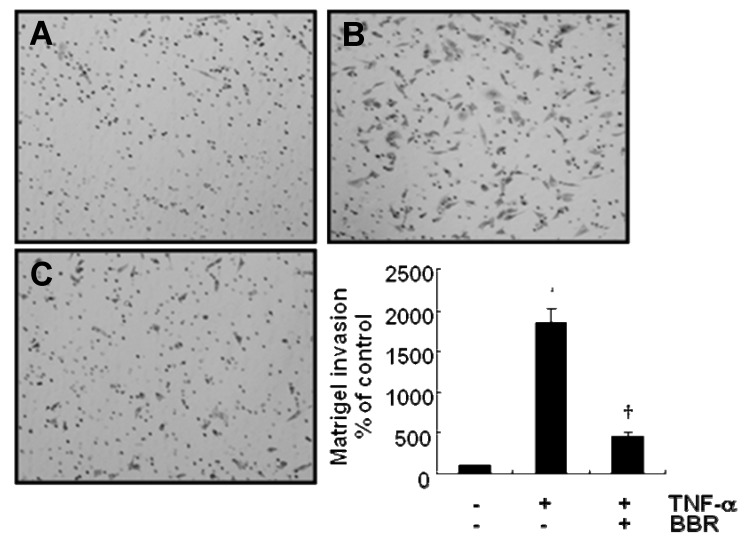
TNF-α-induced cell invasion was suppressed by berberine in MDA-MB-231 cells. After seeding of 1×10^5^ cells/well, cells pretreated with 10 μM berberine for 1 h and then treated with 20 ng/mL TNF-α for 24 h. After 24 h incubation, cells on the bottom side of filter were fixed, stained and conunted. (A) Control; (B) TNF-α alone; (C) TNF-α with berberine. These results were representative of three independent experiments. Values shown are means ± SEM. * *P* < 0.01 *vs*. control, † *P* < 0.01 *vs*. TNF-α-treated cells. Con; control.

Finally, we investigated that BBR suppresses TNF-α-induced MMP-9 expression in MDA-MB-231 cells led us to investigate the effect of BBR on invasion of MDA-MB-231 cells by TNF-α. We pretreated cells with 10 μM BBR prior to 20 μg/mL TNF-α for 1 h and then incubated 24 h. Our results showed that TNF-α significantly increased the invasion of MDA-MB-231 cells (Figure 4). The level of TNF-α-induced cell invasion was significantly increased by 1,843.3±181.9% of control level.However, TNF-α-induced cell invasion was suppressed by BBR treatment (Figure 4). TNF-α-induced cell invasion was greatly decreased by 459.3±52.1% of control level in response to 10 μM BBR treatment. Therefore, we demonstrated that BBR suppresses TNF-α-induced cell invasion through inhibition of MMP-9 expression in MDA-MB-231 cells.

Berberine, one of the major alkaloids, has a wide range of pharmacological effects including inhibition of protein synthesis, cell cycle progression and apoptosis in various cancer cell lines [14,19]. However, little is known about the effects of berberine on tumor invasion in MDA-MB-231 breast cancer cells. Therefore, we investigated the effect of berberine on TNF-α-induced MMP-9 gelatinase activity/expression and cell invasion in MDA-MB-231 cells.

TNF-α regulates tumor remodeling by stimulating fibroblast and macrophage activity, tumor cell motility and tumor invasion through the induction of MMPs [20,21]. TNF-α is also a major cytokine involved in inflammation and immunity [22]. TNF-α expresses in tumor micro-environment of breast, ovarian, colorectal, melanoma and leukemia [23]. Non-contact co-culture of ovarian and breast cancer cells with macrophages increased the invasiveness of tumor cells via TNF-α-dependent activation of JNK and NF-κB pathway [24]. Our results showed that TNF-α dose-dependently increases MMP-9 gelatinase activity/expression in MDA-MB-231 cells. TNF-α-induced MMP-9 gelatinase activity/expression was decreased by MEK inhibitor (UO126) and p38 inhibitor (SB203580). Therefore, we demonstrated that berberine may down-regulate TNF-α-induced MMP-9 gelatinase activity/expression via inhibition of ERK and p38 pathways in MDA-MB-231 cells.

The transcriptional regulation of MMP-9 has been well investigated in various cell types including breast cancer and lung cancer cells. The MMP-9 promoter has several transcription factor-binding motifs, including AP-1 and NF-κB [10]. AP-1 transcriptional factor induces MMP-9 expression in human keratinocytes [18]. Furthermore, MAP kinases including ERK, JNK and p38 are known to be involved in the regulation of MMP-9 transcription [25]. These activated MAP kinases increase the expression and activation of c-Fos and c-Jun, which are the main components of AP-1 transcription factors [25,26]. Activated AP-1 binds to its response element on the MMP-9 promoter to increase the transcription of MMP-9 [25]. In consistent with these reports, our results showed that TNF-α-induced AP-1 DNA binding activity is significantly decreased by berberine. Therefore, we demonstrated that berberine suppresses TNF-α-induced MMP-9 gelatinase activity/expression through inhibition of AP-1 DNA binding activity in MDA-MB-231 cells.

The enhancement of MMPs expression is involved in tumor invasion, metastasis and angiogenesis [27,28]. The inhibition of MMPs expression may play an important role on cancer therapy because MMPs trigger the degradation of extracellular matrix (ECM) and induce tumor invasion [28]. Our results show that TNF-α-induced cell invasion is prevented by berberine treatment in MDA-MB-231 human breast cancer cells. Based on these results, we demonstrated that berberine suppresses TNF-α-induced MMP-9 gelatinase activity/expression and invasion of tumor cell.

## Conclusions

In summary, our results suggest that berberine has novel function to prevent MMP-9-induced degradation of ECM including type IV collagen, leading to inhibition of AP-1 DNA binding activity and suppress cell invasion of MDA-MB-231 cells. Therefore, we suggest that berberine may be used as an effective ingredient for anti-cancer products, which can prevent cancer metastasis and the degradation of ECM proteins by MMP-9.

## Experimental

### Reagents and cell cultures

Cell culture media, antibiotics and 10% Zymogram gel were purchased from Life Technologies, (Rockville, MD). MMP-9 antibody was obtained from Lab vision (Neomarker, Fremont, CA). TNF-α was purchased from R&D Systems (Minneapolis, MN). The secondary peroxidase-conjugated Antibodies and ECL reagents were from Amersham (Buckinghamshire, UK). Berberine was purchased from Sigma (St.Louis, MO). Human breast cancer cell line, MDA-MB-231 cells cultured in Dulbecco’s modified Eagle’s medium (DMEM) supplemented with 10% fetal bovine serum (FBS), 2 mM glutamine, 100 IU/mL penicillin and 100 μg/mL streptomycin. For experiments, MDA-MB-231 cells were maintained in culture medium supplemented without FBS for 16 h.

### MTT assay

The viability of cells was monitored after various concentrations of berberine treatment. 3-(4,5-dimethylthiazol-2-yl)-2,5-diphenyltetrazolium bromide (MTT) was used for the quantification of living metabolically active cells. Mitochondrial dehydrogenases metabolize MTT to a purple formazan dye, which was measured photometrically at 570 nm [17].

### Zymography

Zymography was performed in 10% polyacrylamide gels that had been cast in the presence of gelatin as described previously [17]. Briefly, samples (culture media) were resuspended in loading buffer and run on a 10% SDS-PAGE gel containing 0.5 mg/mL gelatin without prior denaturation. After electrophoresis, gels were washed to remove SDS and incubated for 30 min at room temperature in a renaturing buffer (50 mM Tris, 5 mM CaCl_2_, 0.02% NaN_3_, 1% Triton X-100). In the next step, gels were incubated for 48 h at 37°C in a developing buffer [50 mM Tris-HCl (pH 7.8) 5 mM CaCl_2_, 0.15 M NaCl and 1% Triton X-100]. Gels were subsequently stained with Coomassie Brilliant Blue G-250 and destained in 30% methanol, 10% acetic acid to detect gelatinase secretion. Signal densities were quantified using a desitometric program (Bio 1D; Vilber Lourmat, Marne La Vallec, France).

### Western blotting

Cell lysates and conditioned culture media were used in immunoblot analysis for MMP-9 protein. Proteins were boiled for 5 min in Laemmli sample buffer and electrophoresed in 10% SDS-PAGE gels. Proteins were transferred to PVDF membrane and the membrane was then blocked in 10% Skim milk in TBS with 0.01% Tween-20 for 15 min. The blots were incubated with anti-mouse MMP-9 antibody (1/1,000 dilution) in 1% TBS/T buffer (0.01% Tween 20 in TBS) at 4°C overnight. Blots were washed three times in TBS with 0.01% Tween 20 and subsequently incubated in anti-rabbit peroxidase-conjugated Antibody (1/2,000 dilution) in TBS/T buffer. After 1 h incubation at room temperature, the blots were washed three times and ECL reagents (Amersham Bioscience, Buckinghamshire, UK) were used for development. Signal densities were quantified using a desitometric program (Bio 1D; Vilber Lourmat, Marne La Vallec, France).

### Electrophoretic Mobility Shift Assays (EMSA)

EMSA were performed using a commercial kit (Promega, Madison, WI), according to the manufacturer's instructions. Briefly, AP-1 (5'-CGC TTG ATG CAG CCG GAA-3') consensus oligonucleotides were end-labeled by T4 polynucleotide kinase using [γ-^32^P]ATP (3000 Ci/mmol; Amersham-Pharmacia Biotech. Piscataway, NJ). Binding reactions were performed for 30 min on ice with 5 µg of protein in 20 µL of binding buffer containing 4% glycerol, 20 mM HEPES (pH 7.9), 1 mM MgCl_2_, 0.5 mM EDTA, 0.5 mM DTT, 50 mM NaCl, 10 mM Tris-HCl (pH 7.5) and 50 µg/mL of poly (dI-dC) and 20,000–25,000 dpm of ^32^P-labeled oligonucleotide. DNA-protein complexes wereseparated from unbound oligonucleotide by electrophoresis through 6% DNA retardation gels (Invitrogen, Carlsbad, CA) using 0.5x Tris-borate.

### Cell invasion assay

Matrigel-coated filter inserts (8 μm pore size) that fit into 24-well invasion chambers were obtained from Becton–Dickinson (San Diego, CA). MDA-MB-231 cells to be tested for invasion were resuspended in culture media (1×10^5^ cells/well) and then added to the upper compartment of the invasion chamber in the presence or absence of TNF-α and/or BBR. Culture media (700 μL) were added to the lower compartment of the invasion chamber. The chambers were incubated at 37°C for 24 h. After incubation, the cells on the upper side of the filter were removed using cotton swabs and the bottom filters were fixed and stained (toluene blue, Sigma, St. Louis, MO). The cells that invaded through the Matrigel and were located on the underside of the filter were counted. Four chambers were used per condition. The values obtained were calculated by averaging the total number of cells from four filters.

### Statistical analysis

Statistical significance was determined using the Student’s t-test. Results are presented as means ± SEM. All *p*-value were two-tailed and significance was accepted when *p* was < 0.05.
